# Supplementary material for: Discovery of terahertz-frequency orbitally coupled magnons in a kagome ferromagnet
Source: Sci Adv. 2025 Jul 4;11(27):eadw1182. doi: 10.1126/sciadv.adw1182 (PMC12227069; doi:10.1126/sciadv.adw1182)
Supplement: Supplementary file 1 — Figs. S1 to S7 Notes S1 to S3 [file sciadv.adw1182_sm.pdf]

Science Advances
AAAS

Supplementary Materials for

Discovery of terahertz-frequency orbitally coupled magnons in a kagome ferromagnet

Mengqian Che *et al.*

Corresponding author: Guang-Ming Zhang, gmzhang@tsinghua.edu.cn;
Luyi Yang, luyi-yang@mail.tsinghua.edu.cn

Sci. Adv. **11**, eadw1182 (2025)
DOI: 10.1126/sciadv.adw1182

This PDF file includes:

Figs. S1 to S7
Notes S1 to S3

Supplementary Figures

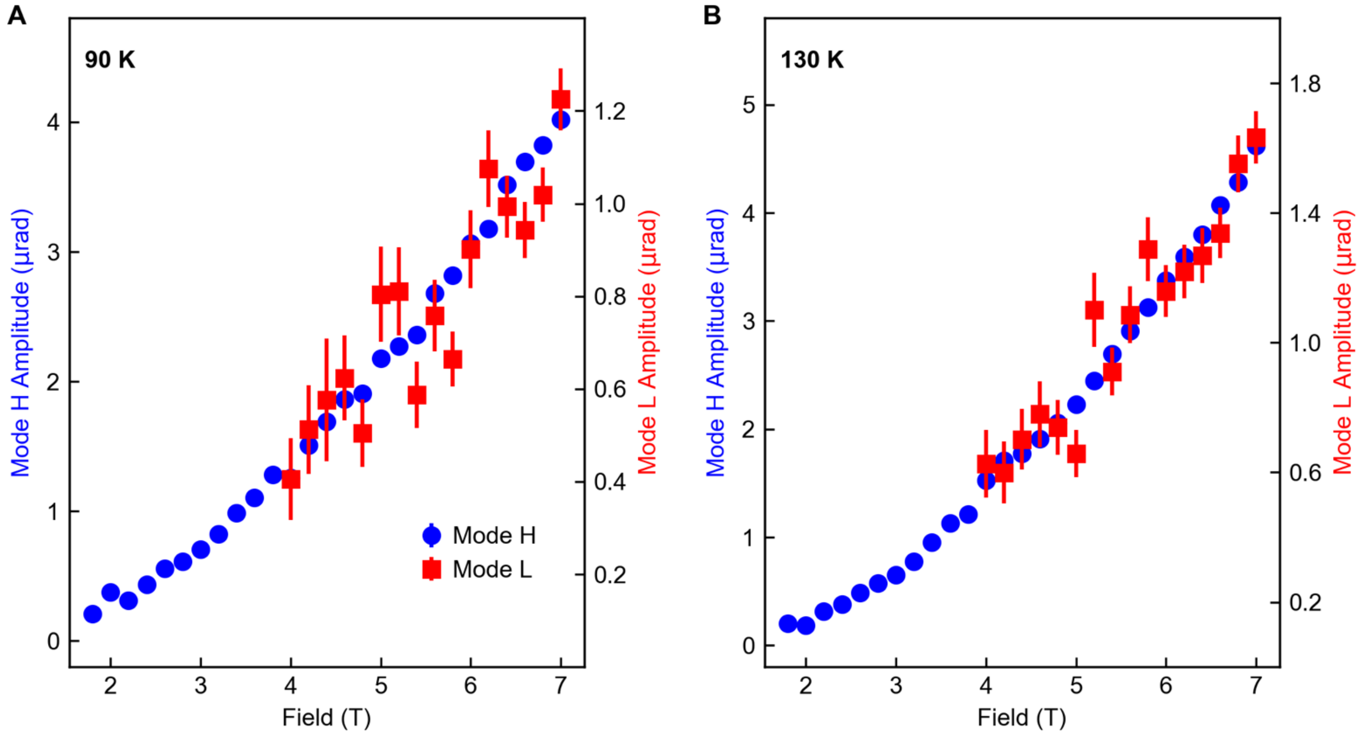

Fig. S1. Mode H and Mode L amplitudes as a function of field. Both amplitudes scale together with the applied field (up to a proportional constant).

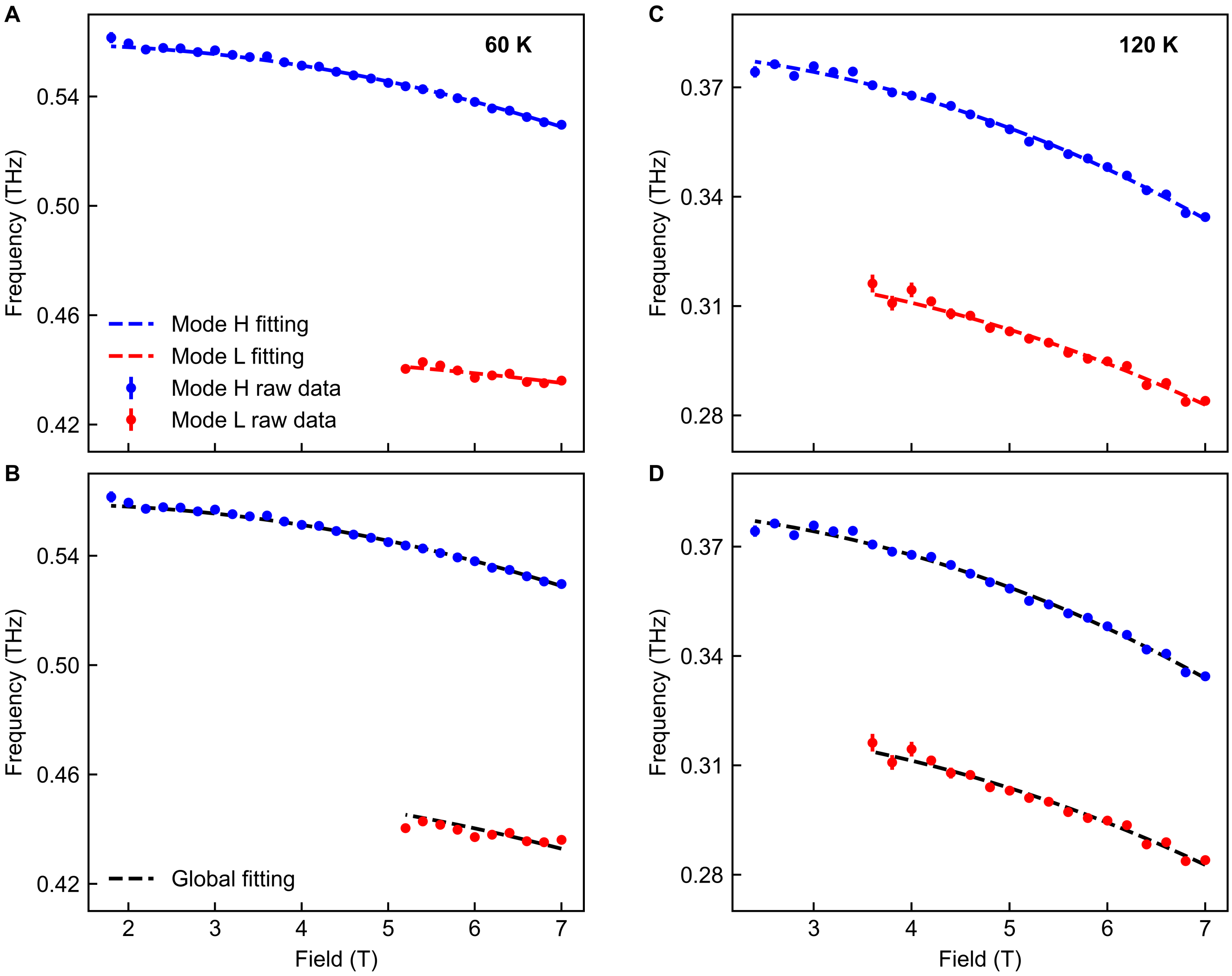

Fig. S2. Fits of the resonant frequency versus field with the Kittel equation. (**A,C**) Individual fitting; (**B,D**) global fitting.

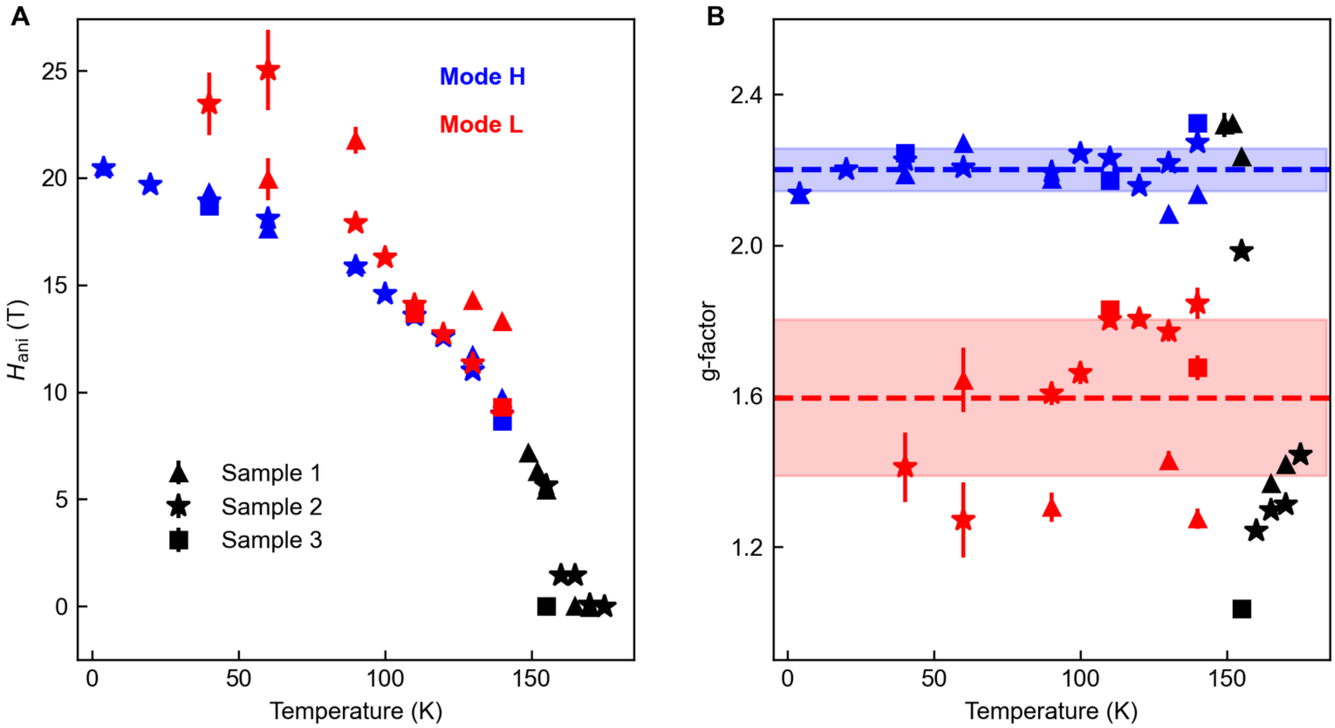

Fig. S3. Anisotropy field (H_{ani}) and Landé g-factor. Similar to Fig. 3A,B (main text), but the data were extracted with individual fitting using the Kittel model. The dashed lines in (**B**) represent the average values of the g-factors, with the shaded areas indicating the associated uncertainties.

Supplementary Note 1: Data Analysis in the Time and Frequency Domains

The raw data in the time domain is modeled using the following equation:

$$\Delta\theta_K(t) = \frac{1 + \mathrm{erf}\,((t - t_0)/\sigma)}{2}\left(\sum_{i=1,2} A_i \cos(\omega_i(t - t_0))\, e^{-\gamma_i(t-t_0)} + \sum_{j=1,2} B_j\, e^{-\Gamma_j(t-t_0)} + C\right). \quad \text{(S1)}$$

The first part represents the instrument's time resolution, characterized by σ, with erf denoting the error function and t_0 representing the time zero. The exponential decaying cosines represent magnetization precession, characterized by amplitude (A_i), frequency ($f_i = \frac{\omega_i}{2\pi}$) and decay rate ($\gamma_i$). Lastly, the background signal is modeled by exponential decays, described by amplitude (B_j) and the decay rate (Γ_j), along with a constant term (C). An example of the time-domain fits is shown in Fig. S4. The obtained instrument's time resolution is 300 fs.

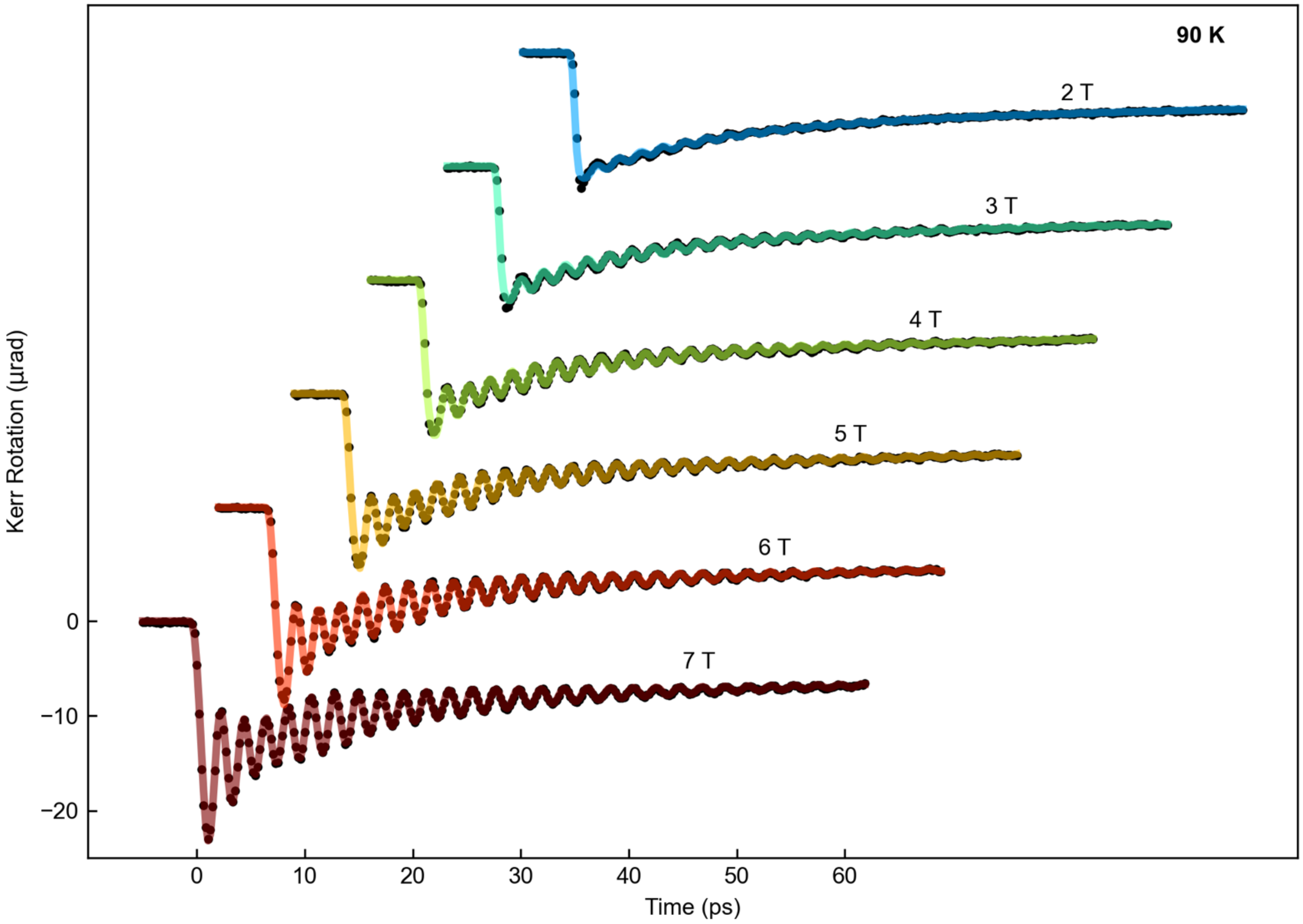

Fig. S4. Fits to the raw data in the time domain using Eq. S1.

Then we subtracted the exponential decay background from the raw data to isolate the oscillatory decaying signal, which was subsequently Fast Fourier transformed to obtain both the real and imaginary parts. An example is shown in Fig. S5.

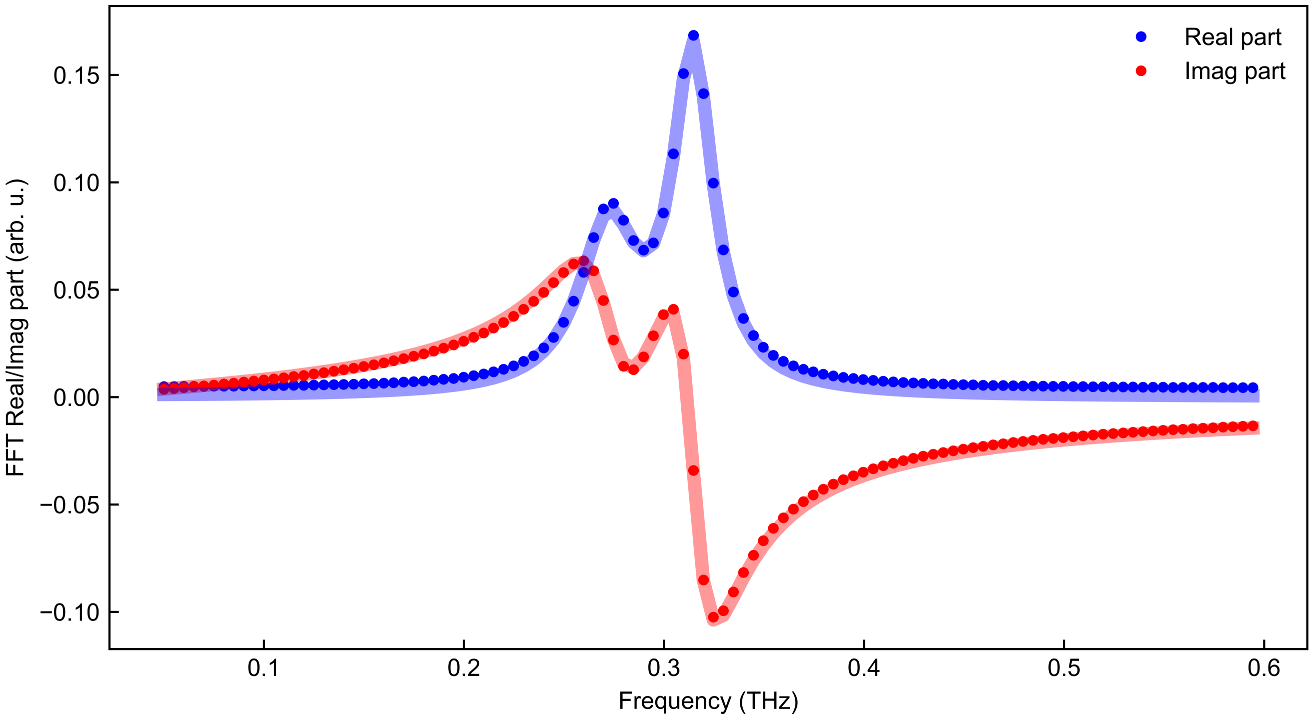

Fig. S5. FFT of oscillatory decaying signals and fits at 130 K and 7 T. FFT Real and imaginary parts of the raw data are illustrated by blue and red dots, respectively. The lines are the fits using Eq. S2. arb. u. stands for arbitrary units.

The FFT real and imaginary parts can be fit simultaneously by the following Lorentzian functions:

$$\begin{cases} A_{real}(\omega) = \dfrac{1}{2\sqrt{2\pi}} \sum\limits_{i=1,2} A_i \left(\dfrac{\gamma_i}{\gamma_i^2 + (\omega - \omega_i)^2} + \dfrac{\gamma_i}{\gamma_i^2 + (\omega + \omega_i)^2} \right) \\ A_{imag}(\omega) = -\dfrac{1}{2\sqrt{2\pi}} \sum\limits_{i=1,2} A_i \left(\dfrac{\omega - \omega_i}{\gamma_i^2 + (\omega - \omega_i)^2} + \dfrac{\omega + \omega_i}{\gamma_i^2 + (\omega + \omega_i)^2} \right) \end{cases}, \quad \text{(S2)}$$

which is basically Fourier transform of two exponentially decaying cosines: $\sum_{i=1,2} A_i \cos(\omega_i t)\, e^{-\gamma_i t}$, $t > 0$ and zero for $t < 0$.

We analyze data in both time and frequency domains to obtain the amplitude (A_i), frequency ($f_i = \frac{\omega_i}{2\pi}$) and lifetime ($\tau_i = \frac{1}{\gamma_i}$) of the magnon modes. Both methods give consistent results in frequencies and

lifetimes. Figure S6 shows an example. More fitted magnon lifetime data at various fields and temperatures are shown in Fig. S7.

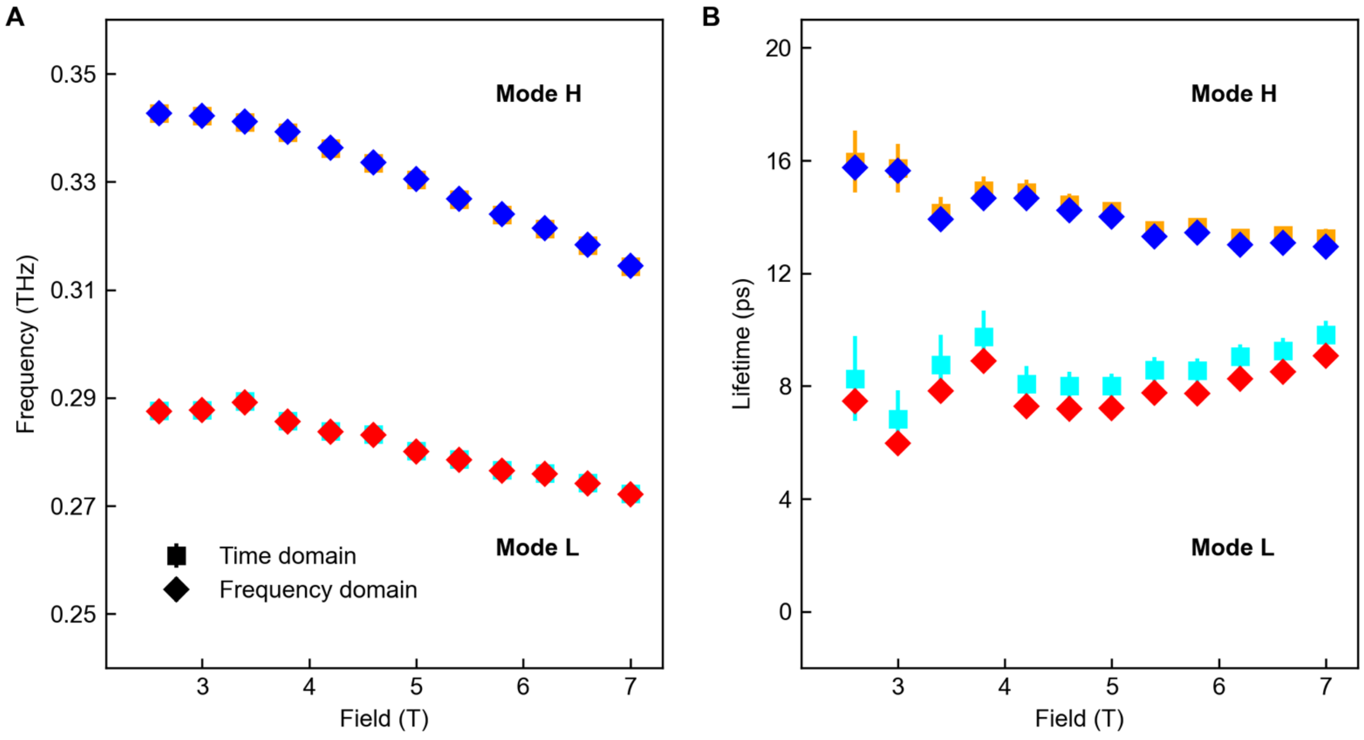

Fig. S6. Fitted magnon frequencies (A) and lifetimes (B) as a function of field from analyses in both time and frequency domains at 130 K.

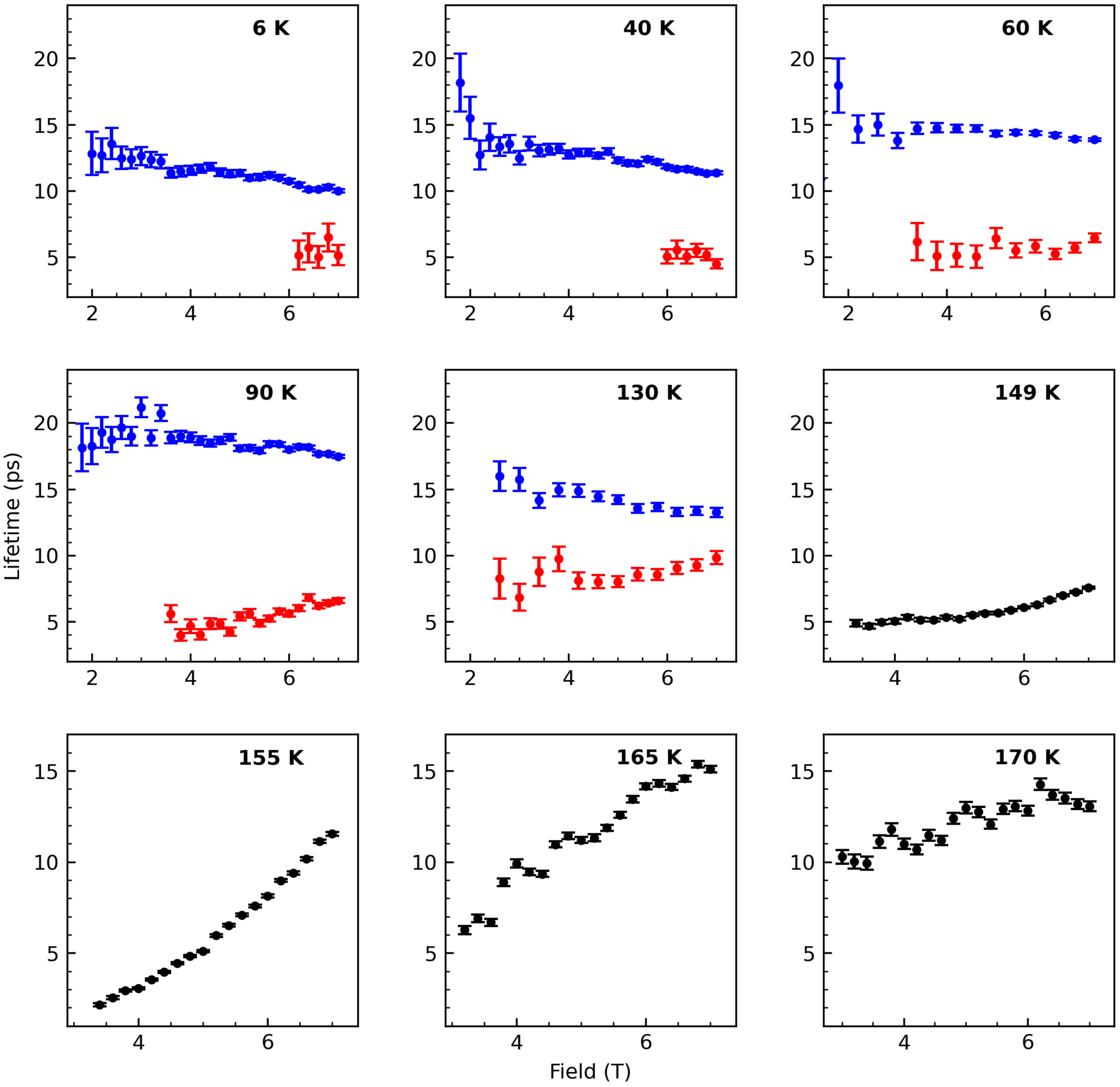

Fig. S7. Magnon lifetimes as a function of magnetic field for various temperatures. At low temperatures, where two distinct modes are observable, Mode H is represented in blue and Mode L is shown in red.

Supplementary Note 2: Calculations of Magnon Modes with the Kittel Model

The ferromagnetic (FM) kagome spin system has three spins per unit cell. The direction of each spin magnetic moment is represented by a unit vector $\hat{S}_i$, with its amplitude denoted by μ_S. We consider both anisotropic Heisenberg exchange coupling and magnetocrystalline anisotropy. The energy of the system per unit cell can be expressed as:

$$U = -\mu_S \left(J_{\text{iso}} \sum_{<ij>} \hat{S}_i \cdot \hat{S}_j + J_{\text{ani}} \sum_{<ij>} \hat{S}_i^z \cdot \hat{S}_j^z + \frac{1}{2} H_K \sum_i \left(\hat{S}_i \cdot \hat{z}\right)^2 + \sum_i \boldsymbol{H}_{\text{ext}} \cdot \hat{S}_i \right) \quad \text{(S3)}$$

where $i = 1,2,3$ denotes the spins within the unit cell and <*ij*> means the summation over the nearest neighbors. $J_{\text{iso}} > 0$ is the dominant isotropic ferromagnetic exchange field, and J_{ani} is the anisotropic exchange field between nearest neighbor spins. H_K and H_{ext} are the magnetocrystalline anisotropic field and the external field strength, respectively. Without any loss of generality, we can set $\boldsymbol{H}_{\text{ext}}$ to be in the x-z plane with an angle θ_H with respect to the z axis.

For static spin configurations, FM coupling leads to spins on different sites parallel to each other. The spin unit vectors at the minimum will then also lie in the x-z plane, and can be represented by a single angle θ_0 with respect to the z axis, reducing the previous equation to

$$U = -3\mu_S \left(2J_{\text{iso}} + 2J_{\text{ani}}(\cos(\theta_0))^2 + \frac{1}{2} H_K (\cos(\theta_0))^2 + H_{\text{ext}} \cos(\theta_H - \theta_0) \right). \quad \text{(S4)}$$

For a given external field, the static position is given by $\frac{\partial U}{\partial \theta_0} = 0$, *i.e.*,

$$H_{\text{ani}} \sin\theta_0 \cos\theta_0 + H_{\text{ext}} \sin(\theta_0 - \theta_H) = 0, \quad \text{(S5)}$$

where $H_{\text{ani}} = H_K + 4J_{\text{ani}}$ is the total anisotropy field.

To calculate the magnon modes, we use the (zero-damping) Landau-Lifshitz-Gilbert (LLG) equation:

$$\frac{d\hat{S}_i}{dt} = -\gamma \hat{S}_i \times \boldsymbol{H}_i^{\text{eff}}, \quad \text{(S6)}$$

where the effective magnetic field is given by

$$\boldsymbol{H}_i^{\text{eff}} = -\frac{\partial U}{\partial \boldsymbol{\mu}_{S,i}} = J_{\text{iso}} \sum_{<j>} \hat{S}_j + J_{\text{ani}} \sum_{<j>} \left(\hat{S}_j \cdot \hat{z}\right)\hat{z} + H_K \left(\hat{S}_i \cdot \hat{z}\right)\hat{z} + \boldsymbol{H}_{\text{ext}}. \quad \text{(S7)}$$

We then linearize the equations around the static spin configuration (1, θ_0, 0) in the spherical coordinate:

$$\frac{d\hat{S}_i}{dt} = d\dot{\theta}_i\hat{\theta}_i + \sin\theta_i d\dot{\phi}_i\hat{\phi}_i. \quad \text{(S8)}$$

The magnon modes and their frequencies can then be obtained from the resulting matrix by calculating the eigenvalues and eigenvectors, which consist of pairs of vectors $\vec{e}_n$ and $\vec{e}_n^*$, with eigenvalues $\pm i\omega_n$. We take the positive eigenvalue (and the corresponding eigenvector) of each pair, the other corresponds to spin precession in the opposite direction. The eigenfrequencies are given by

$$\omega_1 = \gamma\sqrt{AB}, \;\; \omega_{2,3} = \gamma\sqrt{(A + 6J_{\mathrm{iso}})(B + 6J_{\mathrm{iso}} + 6J_{\mathrm{ani}}(\sin\theta_0)^2)}, \quad \text{(S9)}$$

where $A = H_{\mathrm{ext}}\cos(\theta_0 - \theta_H) + H_{\mathrm{ani}}(\cos\theta_0)^2$ and $B = H_{\mathrm{ext}}\cos(\theta_0 - \theta_H) + H_{\mathrm{ani}}\cos(2\theta_0)$. The corresponding eigenvectors in the basis of $\{d\theta_1, d\theta_2, d\theta_3, \sin\theta_0 d\phi_1, \sin\theta_0 d\phi_2, \sin\theta_0 d\phi_3\}$ are given by

$$\vec{e}_1 = \left(\frac{i\sqrt{A}}{\sqrt{B}}, \frac{i\sqrt{A}}{\sqrt{B}}, \frac{i\sqrt{A}}{\sqrt{B}}, 1,1,1\right), \quad \text{(S10)}$$

$$\vec{e}_2 = \left(-\frac{i\sqrt{A + 6J_{\mathrm{iso}}}}{\sqrt{B + 6J_{\mathrm{iso}} + 6J_{\mathrm{ani}}(\sin\theta_0)^2}}, 0, \frac{i\sqrt{A + 6J_{\mathrm{iso}}}}{\sqrt{B + 6J_{\mathrm{iso}} + 6J_{\mathrm{ani}}(\sin\theta_0)^2}}, -1,0,1\right), \quad \text{(S11)}$$

$$\vec{e}_3 = \left(-\frac{i\sqrt{A + 6J_{\mathrm{iso}}}}{\sqrt{B + 6J_{\mathrm{iso}} + 6J_{\mathrm{ani}}(\sin\theta_0)^2}}, \frac{i\sqrt{A + 6J_{\mathrm{iso}}}}{\sqrt{B + 6J_{\mathrm{iso}} + 6J_{\mathrm{ani}}(\sin\theta_0)^2}}, 0, -1,1,0\right). \quad \text{(S12)}$$

Notably, ω_1 is just the Kittel equation (Eq. 1 of the main text) and $\vec{e}_1$ is the acoustic mode with spins on different sites precessing in phase. $\vec{e}_{2,3}$ are higher energy ($\omega_{2,3} > \omega_1$) degenerate optical modes with spin precession out of phase, resulting in zero net magnetization change with time. Since our probe is sensitive to the total magnetization change along the out-of-plane direction ($\Delta M_z(t)$), only the acoustic mode is detectable in our experiment. At $H_{\mathrm{ext}} = 0$ or high field limit $H_{\mathrm{ext}} \gg H_{\mathrm{ani}}$, $A \cong B$ and all spins precess in a circular motion; otherwise, the motion is elliptical.

Supplementary Note 3: Coupled Spin-Orbital Model

We further consider a coupled spin and orbital system with isotropic Heisenberg exchange coupling for simplicity, noting that extending this to the anisotropic case is straightforward. The directions of each spin and orbital magnetic moment are represented by a unit vector $\hat{S}_i$ and $\hat{L}_i$ with magnitudes denoted by μ_S and μ_L, respectively. The energy of the system per unit cell can be expressed as:

$$U = -\mu_S\left(J\sum_{<ij>}\hat{S}_i\cdot\hat{S}_j + \frac{1}{2}H_{KS}\sum_i\left(\hat{S}_i\cdot\hat{z}\right)^2 + \sum_i \boldsymbol{H}_{\text{ext}}\cdot\hat{S}_i\right) - \mu_L\left(Q\sum_{<ij>}\hat{L}_i\cdot\hat{L}_j + \frac{1}{2}H_{KL}\sum_i\left(\hat{L}_i\cdot\hat{z}\right)^2 - \sum_i \boldsymbol{H}_{\text{ext}}\cdot\hat{L}_i\right) - \frac{\lambda}{\mu_B}\mu_S\mu_L\sum_i\hat{S}_i\cdot\hat{L}_i\,, \qquad \text{(S13)}$$

where <*ij*> means the summation over the nearest neighbor lattice sites. *J*, *Q* > 0 is the isotropic FM exchange field; H_{KS} and H_{KL} are the magnetocrystalline anisotropic fields; the Zeeman terms have opposite signs for spin and orbital magnetic moments, reflecting the diamagnetic property of orbital magnetic moments observed experimentally (26,28); λ characterizes the coupling between spin and orbital magnetic moments.

Similar to the discussion in Supplementary Note 2, we can set $\boldsymbol{H}_{\text{ext}}$ to be in the x-z plane with an angle θ_H with respect to the z axis. Then the equilibrium positions of $\hat{S}_i$ and $\hat{L}_i$ are also in the x-z plane with an angle $\{\theta_{S,i}, \theta_{L,i}\}$ with respect to the z axis. These angles are determined by minimizing *U* with respect to $\theta_{S,i}$ *and* $\theta_{L,i}$, respectively:

$$\frac{\partial U}{\partial\theta_{S,i}} = \mu_S\left(J\sum_{<j>}\sin(\theta_i-\theta_j) + \frac{1}{2}H_{KS}\sin\left(2\theta_{S,i}\right) + H_{\text{ext}}\sin\left(\theta_{S,i}-\theta_H\right) + \frac{\lambda}{\mu_B}\mu_L\sin\left(\theta_{S,i}-\theta_{L,i}\right)\right) = 0, \qquad \text{(S14)}$$

$$\frac{\partial U}{\partial\theta_{L,i}} = \mu_L\left(Q\sum_{<j>}\sin(\theta_i-\theta_j) + \frac{1}{2}H_{KL}\sin\left(2\theta_{L,i}\right) - H_{\text{ext}}\sin\left(\theta_{L,i}-\theta_H\right) + \frac{\lambda}{\mu_B}\mu_S\sin\left(\theta_{L,i}-\theta_{S,i}\right)\right) = 0, \qquad \text{(S15)}$$

To calculate the magnon modes, we use the (zero-damping) LLG equation:

$$\frac{d\hat{S}_i}{dt} = -\gamma_S\hat{S}_i\times\boldsymbol{H}_{S,i}^{\text{eff}}, \qquad \text{(S16)}$$

$$\frac{d\hat{L}_i}{dt} = -\gamma_L \hat{L}_i \times \boldsymbol{H}_{L,i}^{\mathrm{eff}}, \tag{S17}$$

where the gyromagnetic ratio $\gamma_\eta = \frac{\mu_B}{\hbar} g_\eta$ with $\eta = S$ or L, and $g_S = 2$ and $g_L = 1$, and the effective field is given by

$$\boldsymbol{H}_{S,i}^{\mathrm{eff}} = -\frac{\partial U}{\partial \boldsymbol{\mu}_{S,i}} = J \sum_{<j>} \hat{S}_j + \boldsymbol{H}_{\mathrm{ext}} + H_{KS}(\hat{S}_i \cdot \hat{z})\hat{z} + \frac{\lambda}{\mu_B} \mu_L \hat{L}_i, \tag{S18}$$

$$\boldsymbol{H}_{L,i}^{\mathrm{eff}} = -\frac{\partial U}{\partial \boldsymbol{\mu}_{L,i}} = Q \sum_{<j>} \hat{L}_j - \boldsymbol{H}_{\mathrm{ext}} + H_{KL}(\hat{L}_i \cdot \hat{z})\hat{z} + \frac{\lambda}{\mu_B} \mu_S \hat{S}_i. \tag{S19}$$

Following similar procedures as outlined in Supplementary Note 2, we obtain eigenfrequencies and eigenvectors. In this coupled spin and orbital system, there are two acoustic magnon modes, corresponding to Mode H and Mode L, and four optical magnon modes with magnetic moments on different sites precessing out of phase with each other. Notably, the frequencies of the acoustic modes are independent of J and Q, but depend on all other parameters. However, the optical modes are not detectable in our experiment due to the cancellation of the net magnetization change. Then we can simplifier our model by removing the exchange coupling terms in the model. After fitting our experimental data with our coupled spin-orbital model, we obtain reasonable fitting parameters as shown in Fig. 4 of the main text.

Without the spin-orbital coupling term, Eq. S13 describes two decoupled spin and orbital systems. In this scenario, the magnetic moment of each evolves independently according to the LLG equation, with the resonance frequency determined by the anisotropy field (zero external field case) and the respective g-factor (2 for spin and 1 for orbital). However, the orbital magnetic moment (0.007μ_B) is too small to produce measurable signals.

In the presence of spin-orbital coupling, the spin magnetic moment strongly interacts with the orbital magnetic moment. This interaction is a pivotal for enabling the observation of the second magnon mode. The eigenvectors of both modes contain both spin and orbital components, denoted by $d\theta_S^M$ and $d\theta_L^M$, respectively, where the superscript *M* represents Mode H or Mode L.

The magnon excitation amplitude is related to the generation mechanism. As described in the main text and Fig. 1B, the pump pulse induces an abrupt alteration in the anisotropy energy owing to the sudden heating of the lattice. Because magnetization states will still be in their old equilibrium positions, this

creates an initial offset from the equilibrium positions, proportional to how the equilibrium position changes. Assuming the same laser induced anisotropy change for spin and orbital (δH_K) and differentiating the equilibrium equations (S14 and S15), we obtain the initial offsets of the effective fields with respect to the equilibrium positions $\delta\theta_\eta$ with $\eta = S$ or L.

The measured magnon amplitude for each mode ($\Delta\theta_{\mathrm{Kerr}}^{M}$) is proportional to

$$\mu_S \sin(\theta_S)\, \delta\theta_S d\theta_S^M + a\mu_L \sin(\theta_L)\, \delta\theta_L d\theta_L^M, \qquad \text{(S20)}$$

where a represents the Kerr rotation sensitivity difference between spin and orbital magnetic moments and is taken as 1 for simplicity, and $\sin(\theta_\eta)$ accounts for the projection of the magnetic moment change on the measurement axis. Given that $\mu_S \gg \mu_L$, the measured signals for both modes are predominantly influenced by the spin magnon component. We use Eq. S20 to fit the magnon amplitude shown in Fig. 1G (main text), and calculate the Kerr rotation amplitude ratio of Mode H over Mode L ($\Delta\theta_{\mathrm{Kerr}}^{\mathrm{Mode\ H}}/\Delta\theta_{\mathrm{Kerr}}^{\mathrm{Mode\ L}}$) as a function of magnetic field and temperature, respectively, shown in Fig. 4E,F (main text). We find excellent agreement between our model and experimental data.
